# Which students skip school? A comparative study of sociodemographic factors and student absenteeism using PISA data

**DOI:** 10.1371/journal.pone.0300537

**Published:** 2024-05-22

**Authors:** Ulf Fredriksson, Maria Rasmusson, Åsa Backlund, Joakim Isaksson, Susanne Kreitz-Sandberg

**Affiliations:** 1 Department of Education, Stockholm University, Stockholm, Sweden; 2 Department of Education, Uppsala University, Uppsala, Sweden; 3 Department of Social Work, Stockholm University, Stockholm, Sweden; University Putra Malaysia: Universiti Putra Malaysia, MALAYSIA

## Abstract

This article explores which students–with regard to gender, socio-economic background and migration background–skip school in Germany, Japan, Sweden and the United Kingdom (UK) according to PISA data. Students who skip school are observed in many countries, but there is not much systematic research that studies this across countries. Comparable data is to a large extent missing. PISA data offers an opportunity to use comparable data. In PISA, students were asked in 2018, 2015 and 2012 whether they had skipped school a whole day in the last two weeks prior to their completion of the PISA student questionnaire. Patterns of how absence relates to sociodemographic factors vary in countries and school systems. In the comparison between the four countries the UK stands out as having a higher percentage of students who have reported that they have skipped school than in the other countries. This does not seem to be related to any specific group of students. Japan also stands out with a lower percentage of students who have reported that they have skipped school. According to PISA data, skipping school is more related to socio-economic background than any other of the variables studied. The socio-economic background seems to be related to skipping school in all three PISA studies in Sweden and the UK. Gender seems not to be an important factor in the four countries. In Sweden and Germany there is a lower percentage of non-immigrant students who report that they have skipped school than first-and second-generation immigrant students. In the UK the figures are more ambiguous. When the percentages of students skipping school are compared over time and in the countries, it is difficult to find any trends, but the data only covered three measurements during a period of six years, which may be too short a time span to see trends.

## 1. Introduction

During recent years the issue of students´ absence from school has been increasingly discussed. Most societies offer education free of charge because it is assumed that education helps children and young persons to develop both their cognitive and their social skills. Education is presented as a right in the Universal Declaration of Human Rights [1] and the Convention of the Right of the Child [2]. It is generally assumed that students’ absence from school has a negative impact on students´ school performance, well-being and future opportunities in life (see for example Maynard et al. [3]).

When absence is discussed, the issue is usually not on authorised absence for a few days caused by, for example, illness, but absence from school for long periods of time that may influence students´ sense of belonging to school. This phenomenon with problematic absence from schools has been observed in many countries [4, 5]. In the US and Japan there have been extensive discussions on school attendance over decades. In some countries, such as Sweden, discussions have only recently become more widespread [6].

As the interest for school attendance problems has increased in recent years it is also of interest to see whether there have been changes over time. Many prevention strategies build on a wish to solve or at least to diminish school problems such as absenteeism. Time line studies in the US have shown that the phenomenon changes little over time although there have been major attempts for prevention strategies [3].

As students’ absence has been observed in many countries it is of growing interest to further research this phenomenon. Much of the research have focused on similar factors that may play a major role in adolescents’ risks of suffering from school attendance problems. There is a lack of systematic studies that shed light on these questions from an international comparative perspective. Comparative education approaches do not only allow an analysis of educational similarities and differences between two or more countries or regions, but also to investigate theoretical understandings and assumptions on education realities [7]. Comparisons between countries could make it possible to understand what may be unique factors behind absenteeism in one country and what may be general factors behind absenteeism in many countries. It is from this perspective interesting to compare selected countries and explore whether there are differences in the national patterns. This article will focus on the sociodemographic factors of gender, socio-economic status (SES) and migration background and explore their relationship to students’ self-reported data on skipping school in different countries. By comparing countries, it may be possible to see what could be global patterns and what is more specific in different national contexts.

A challenge when comparing school absenteeism between countries is that comparable data is–despite the broad literature on school attendance problems–to a large extent missing [8, 9]. School absence is in different countries defined in various ways, and registered in diverse ways, and the information collected is made available in different ways. It is therefore difficult to compare available national statistics. This article will try to analyse data on school absence using a large-scale international comparative study. In the Programme for International Student Assessment (PISA), which focus on students’ school achievements, such data on school absence is available. PISA, which is often described as the largest educational study in the world, offer some possibilities to compare student absenteeism in schools between countries (see Section 4: Methods and study assumptions below). Absenteeism is sometimes referred to as one of many variables when PISA data are analysed, but more seldom with a focus on absenteeism *per se*. Even though PISA data could be used to make international comparisons concerning student absenteeism there are few published studies where this has been done in a more specific way. Among the few studies where PISA data have been used earlier for the analysis of truancy, the study by Keppens & Spruyt [10] and Fredriksson et al, [11] can be found. Keppen´s and Spruyt’s [10] analysed European countries that participated in the PISA study 2012 to explore to what degree truancy rates varied between countries and if those rates related to characteristics of the educational systems. Fredriksson et al. [11] mainly showed that PISA data could be used for a comparison between some countries concerning the percentage of students skipping school and trends in the countries. In this article, we will look more in detail into how the results for truancy in the PISA data can be used to understand students’ school absences in some selected countries over the time-spam of three consecutive PISA studies.

## 2. Aim of the article

The aim of this article is to explore which students who skip school with regard to gender, socio-economic background and migration background in Germany, Japan, Sweden and the United Kingdom (UK) according to PISA data. These four countries have been chosen to reflect different school systems and welfare states in a larger project looking at school attendance problems from an international comparative perspective. Sweden is included as the group of researchers involved in the project are based in Sweden. Germany and the UK are important countries in the European context with many links to Sweden but with different school and welfare state systems. Japan is of interest as school absenteeism is a topic that has been discussed in the country for many years (see for example Horiguchi [12], Yoneyama, [13]). It can also be noted that all four countries, as member states of the OECD, belong to wealthier states in the world.

We will focus on what can be described as sociodemographic variables. More specifically, students who skip school will be explored in relation to gender, socio-economic background and migration background. These three variables can be regarded as variables describing social and cultural background characteristics of the students, but they are also variables which are relevant in relation to educational and social inequality and the particularly close connection between social background and school achievement. It can therefore be relevant to do a comparison between countries. Data on these variables are available from the PISA studies. Age, which is often used in similar contexts, is not relevant to use when PISA data are used as all participants are of the same age. We note that in studies from the US references are often made to race and ethnicity, but there is no variable in PISA that corresponds to this. Whether there are differences between the countries in respect of the percentage of students absent from school may be relevant for further discussions on differences in educational as well as welfare systems.

The following questions are asked:

- Which are the proportions of students who skip school in respect of gender, socio-economic background and migration background?
- Are there differences or similarities in patterns between the countries in respect of the sociodemographic background of students who reported they have skipped school?
- Is it possible to observe any trends concerning the composition of the group of students who skipped school in the different countries?

## 3. The multi-dimensional field of school attendance research

The definitions of school attendance problems that is used in recent literature, differentiates between four major forms of school attendance problems: school refusal, school truancy, school withdrawal and school expulsion [14]. The terms are however also overlapping [15, 16] and Kearney argues that “school attendance problems have no consensus definition […] but lack of school attendance as well as permanent school dropout have been identified as widespread global phenomena” [16 p. 2]. Short and sporadic periods of absence are investigated in terms of truancy [17]. Truancy has been described as a gradual process of school disengagement [10].

Most researchers would agree that contextual factors play a major role in adolescents´ risks of suffering from school attendance problems. Kearney and Gonzálvez [18] have thematized contextual risk factors for school attendance problems. According to Ekstrand [19] the factors which are assumed to influence truancy are often sorted under four headings: individual, family, school and society. Maynard et al. [3] analysed truancy based on sociodemographic factors, substance use and family factors. To analyse the sociodemographic factors, Maynard et al. [3] used the variables age, gender and race/ethnicity. There are several studies which have tried to look at these factors in Western societies [10, 20, 21] but also in Japan absenteeism has been described as more frequent for economically and socially disadvantaged groups [12, 22–24].

Harakeh et al. [25] identified gender as one, among many factors, that could predict truancy among 15- to 16-year olds. According to Ekstrand [19] gender, in much research, has been identified as a variable that influence truancy (see, for example, Attwood & Croll [26]). In another study it seems that girls are found as often as boys in the statistics of unauthorized absence [27]. Studies on the influence of gender have, according to Maynard et al. [3], been less disclosive. Also, Gubbels et al. [28] note that most of the risk domains they had identified in their literature review were not moderated by gender. They draw the conclusions that this indicates that the effect of most risk domains for school absenteeism seem similar for boys and girls.

Maynard et al. [3] studies in the US have illustrated the influence of race, age and gender on truancy. They argue with the support of Vaughn et al. [21] that most truant youths participating in the least amount of skipping school were white. Those who moderately skipped school were either African American or Hispanic and those who frequently skipped school were evenly split between White, African American, and Hispanic youths. In many European studies, it is more usual to refer to the influence of migration background and its interaction with other dimensions of social inequality than to race. Rademacker [29] discussed for example how in the process of reproducing inequalities in the society and the education system, stigmatisation of children with migration background may also be reflected in the reality of school attendance. Keppens and Spruyt, [10] showed that ethnicity, operationalised in relation to first and second generation-migration background, correlated with truancy.

In addition to age, gender and race/ethnicity, risk factors for absenteeism are sometimes described in line with psychological dimensions [30]. Gubbels et al. [28] found in their systematic literature review that students’ school absenteeism was associated with issues related to physical and mental problems of the child, substance abuse, antisocial or risky behaviour, problems at or with school, characteristics of the school, parenting problems and difficulties and family problems. Other studies show that school organization can play an important role, both for inducing and for solving the problem [31–33]. In a study of upper secondary schools in Stockholm (Sweden) it was shown that schools in which the teachers rated the leadership and the schools´ ethos as high, students were significantly less likely to report truancy [34]. Karlberg et al. [20] came to similar conclusions in a study of 101 Swedish schools, where they found an association between positive school climate and lower unauthorized absenteeism in students’ ratings. In other words, school attendance problems appear to be an internationally well-researched, multi-dimensional field and this article will not touch on all sub-fields and explanation models but only investigate how sociodemographic factors relate to school attendance. It should also be noted that this is a phenomenon that have been referred to in different ways. As this study will use PISA data it will mainly refer to students who have skipped school as this is the questions students have responded to (see section 4: Methods and study assumptions below),

## 4. Methods and study assumptions

Based on the literature referred to above some assumptions can be made and the following hypothesis will guide our analysis and discussion:

1. As countries are different regarding socio-economic conditions, education systems, welfare state models, and other aspects, there may be differences in rates of students skipping school between different countries.
2. As gender patterns related to school absence are inconsistent according to the international literature, it could be assumed that we find different pattern between boys and girls in the studied countries.
3. As socio-economic background seems to influence many aspects of education, it seems likely that it also influences rates of students skipping school in different countries.
4. As we know from other comparisons between non-migrant and migrant students, those who have lived longer in respective countries seem to do better in school. This may also be reflected in the rates of students skipping school.
5. As shown by American research absence rates seem to be a phenomenon that is difficult to change over time, which may suggest that we can expect no or only little change between different studies (2012, 2015 and 2018).

We will use PISA data, as we have used this data earlier in a study to explore what data PISA contained that could be used for further comparative analysis of students’ school absence in Germany, Japan, Sweden and the UK (listed in alphabetical order) [11]. In PISA 2018 about 600,0000 15-year old students participated from 79 countries. As PISA is based on a representative sample of students these 600,000 students are supposed to represent 32 million 15-year old students in the 79 participating countries [35]. An earlier study has explored what data PISA contains that could be used for further comparative analysis of students’ school absence and how this data could be used [11].

The student questionnaire in PISA 2018 contained the following question (see S1 Appendix for the translations into German, Japanese and Swedish):

*In the last two full weeks of school, how often did the following things occur?*

- *I skipped a whole school day*.
- *I skipped some classes*.
- *I arrived late for school*.

For each statement the student could choose between the following responses:

*Never; One or two times; Three or four times; Five or more times*

It is possible to look at students’ school self-reported absence based on the same question put to the students for the years 2018, 2015 and 2012. It is also possible to get information about absenteeism for the year 2000, but as the question was partly phrased in a different way in PISA 2000, this year will not be included in this article.

The measure we use, based on PISA, is that students who have reported that they have skipped school at least one day during the two weeks prior to the PISA test are defined as absent students. It should be noted, as mentioned in section 3: The multi-dimensional field of school attendance research, that there are many terms and definitions used in relation to school absence problems. The measure used in PISA has a higher threshold level than Maynard et al. [3], who refer to skipping school at least once during the previous month, but lower than the definition used by Kearny [15], who put the threshold at 2.5 days during a period of two weeks. In this definition we assume to be able to capture sporadic truancy, but possibly also other categories of school attendance problems, as school refusal. As truancy has been described as a gradual process of school disengagement [10], it makes sense to investigate statistics on short-term absence, such as the PISA data, for the study of school absenteeism.

In addition to the specific data on students skipping school PISA also contains data from the student questionnaires which can be combined with the data about absence. Data on absence can be related to data on students’ characteristics such as gender, socio-economic background and migration background.

PISA contains data from the four countries of interest in this article (Germany, Japan, Sweden and the UK). These four countries have participated in all the PISA studies since the year 2000. This means that it is possible to look at self-reported absence and student background in all four countries over the period 2012–2018.

The article has used OECD´s international PISA database with data from all PISA studies and from all participating countries [36]. This is a public database provided by the OECD. The database does not contain any links to other databases. Individual students cannot be identified or linked to data in other databases. The collection of data in the PISA project has been approved by the governments concerned and made public in the OECD database. Issues related to the consent of the participants and information to them have been dealt with by each country participating in PISA as part of their data collection [37].

The data analysed students’ self-declared absence, gender, socio-economic status and migration background from the student questionnaire. How socio-economic status and migration background are defined will be explained in section 6.2: Socio-economic differences and section 6.3: Migration background covering the specific topics. More information about PISA data is available in the PISA 2018 Technical Report [37]. Data is analysed to examine differences between the countries included in the study and time trends in the countries. Data has been identified in the database and relevant calculations made. The demands of PISA’s complex survey design have been considered, and appropriate weights and methods of analysis [38] have been used. The analysis utilized version 5.0.13 of the International Database Analyzer (IDB) [39] to calculate the prevalence of school absenteeism among students and the three student subgroups within each country, that is gender, socio-economic status, and migration background. Additionally, the software was employed to estimate confidence intervals used for significance testing. The analyses consist of descriptive statistics, significance tests of differences and logistic regression.

In the logistic regression models, a binary outcome variable is used. It is coded as 1 when students have responded that they have skipped school one day or more during the last two weeks and 0 when students did not skip school at all. The PISA index for economic, social, and cultural status (ESCS) is a continuous variable. The variable for immigrant background has three values: non-immigration, first- and second-generation immigrants. This variable was transformed into two dummy variables for non-immigration background or second-generation background. The gender variable was coded 1 for female students. The IDB (International Database Analyzer) version 5.0.13 [39] was used for the regression analyses, and that ensures that sampling weights are always used and standard errors are correctly computed using the required Balanced Repeated Replication (BBR) method.

## 5. Number of students in PISA and the proportion that have skipped school

Each country participating in PISA has to make a representative sample of 15-year-old students. For more detailed information about how the samples have been made, see OECD [37]. Table 1 presents the number of students included in the samples from the four countries covered in this article. As mentioned in Section 4: Methods and study assumptions, there were no questions about absence in the PISA studies in 2009, 2006 and 2003 and the question was partly differently phrased in 2000. These years will not be dealt with in the further analysis and are not presented in the table below.

**Table 1:** Number of students in the different national samples.

Country / Year	Germany	Japan	Sweden	UK	Total
2012	5,001	6,351	4,736	12,659	28,747
2015	6,504	6,647	5,458	14,157	32,766
2018	5,451	6,109	5,504	13,818	30,882

These samples are sufficiently big and made in such a way that it is possible to generalise for the whole population of 15-year olds in the respective country.

Table 2 shows the number and the percentage of students who have reported that they have skipped a whole school day at least once during the last two full weeks of school before they completed the PISA student questionnaire.

**Table 2:** Number and percentage of all students in each country (who answered this question) who reported that they have skipped a whole school day at least once during the last two full weeks of school before they completed the PISA student questionnaire.

Country Year	Germany		Japan		Sweden		UK	
	n	%	n	%	n	%	n	%
2012	218	4.4	98	1.5	334	7.0	2,607	17.6
2015	474	7.2	115	1.8	465	8.6	3,762	24.4
2018	327	6.0	123	2.1	475	8.8	2,836	17.0

As can be seen in the table, it concerns some hundred students, with the exception of the UK, where the numbers are considerably higher. This is partly related to the fact that the UK samples, as seen in Table 1, are larger.

As the size of the samples has varied between years and between countries the comparison of percentages between the countries seems to be the most appropriate way of analysing the data. It can be seen that the UK seems to have a higher percentage of students who have skipped school than in the other countries and Japan seems to have a smaller percentage (see also Fredriksson et al. [11]).

## 6. Sociodemographic factors and school attendance

The next step in the analysis is to look at how different groups of students reported in the PISA student questionnaire about skipping school at least one day during the two weeks prior to their completing the questionnaire. A more detailed picture will be given by using self-reported data from the PISA student questionnaires about gender, socio-economic background and migration background.

For all the years, in all four countries, there were significant differences (p ≤ .05) in all the categories studied (boys, girls, students in the first quartile of the ESCS index, students in the fourth quartile of the ESCS index, non-immigrant students, first generation immigrant students and second generation immigrant students) between the percentages of students who had been absent at least one day from school during the two weeks before the PISA test and the students who had not been absent at all.

### 6.1. Gender differences

Table 3 shows the number and percentage of boys and girls who reported in the PISA student questionnaire that they have skipped at least one full school day during the two weeks before they completed the questionnaire as a percentage of the boys and girls, respectively, that answered the question about skipping school in each country.

**Table 3:** Number and percentage of boys and girls who have reported that they have skipped school at least one day during the two weeks before the PISA-test.

*Country/year*	*Germany*		*Japan*		*Sweden*		*UK*	
	*Boys*	*Girls*	*Boys*	*Girls*	*Boys*	*Girls*	*Boys*	*Girls*
	*%*	*%*	*%*	*%*	*%*	*%*	*%*	*%*
	*(n)*	*(n)*	*(n)*	*(n)*	*(n)*	*(n)*	*(n)*	*(n)*
*2012*	*4*,*4 (107)*	*4*.*5 (111)*	*1*.*8 (64)*	*1*.*2 (34)*	*6*.*9 (165)*	*7*.*1 (169)*	*16*.*0 (1244)*	*19*.*1 (1363)*
*2015*	*6*.*6 (223)*	*7*.*8 (251)*	*2*.*1 (67)*	*1*.*4 (48)*	*8*.*3 (230)*	*8*.*9 (235)*	*23*.*1 (1851)*	*25*.*7 (1911)*
*2018*	*6*.*8 (194)*	*5*.*1 (133)*	*2*.*3 (65)*	*1*.*9 (58)*	*9*.*5 (250)*	*8*.*1 (225)*	*16*.*9 (1399)*	*17*.*1 (1437)*

Note. The proportion of boys/girls refers to the subset of male/female respondents who provided responses regarding skipping m school for at least one day.

As was shown in Section 5:—Number of students in PISA and the proportion that have skipped school, there are more students in the UK, both in absolute numbers and in percentage, who reported that they have skipped school. This does not change when the students are split between boys and girls. There are both more girls and boys in the UK who report that they have skipped school than in any of the other three countries. In Japan, there are fewer students, both boys and girls, who reported that they have skipped school than in the other countries.

**Fig 1:**
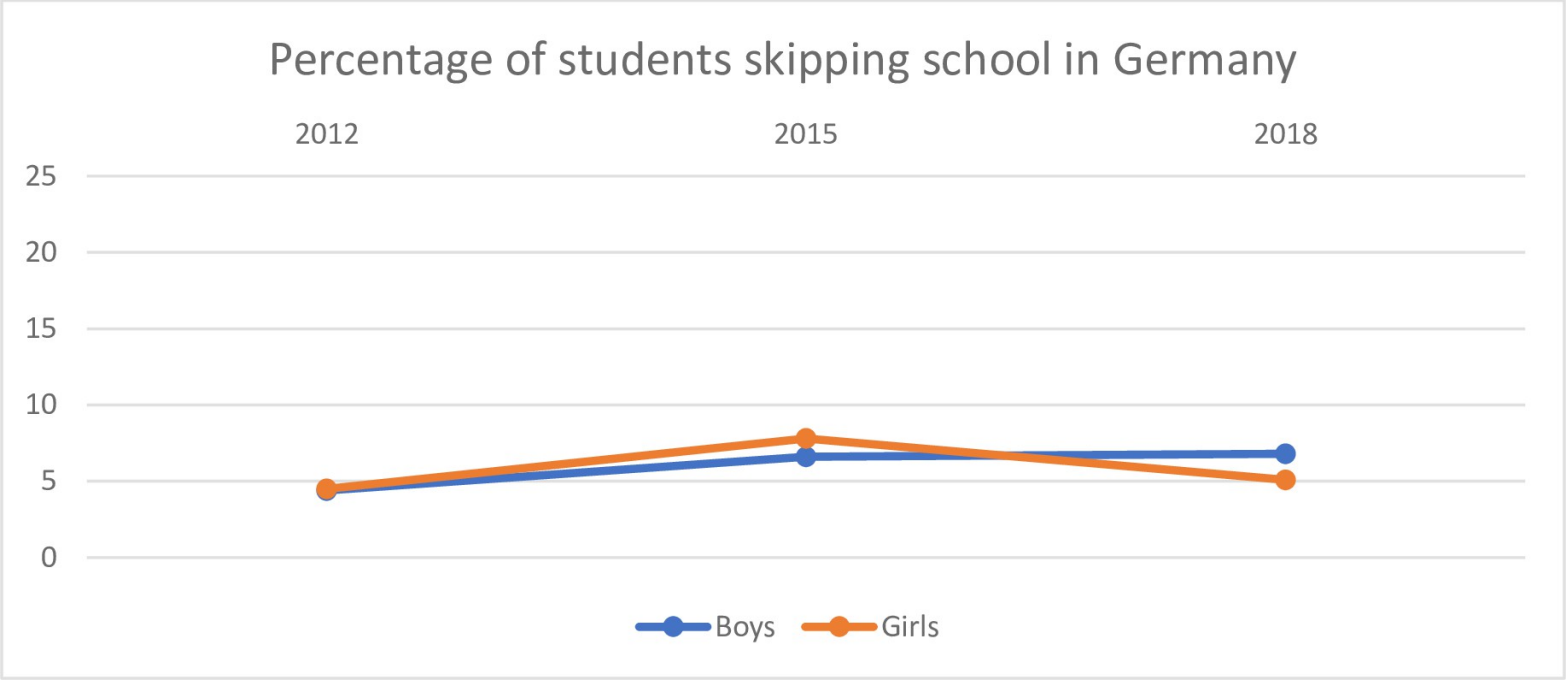
Percentage of boys and girls in Germany who have reported that they skipped school.

**Fig 2:**
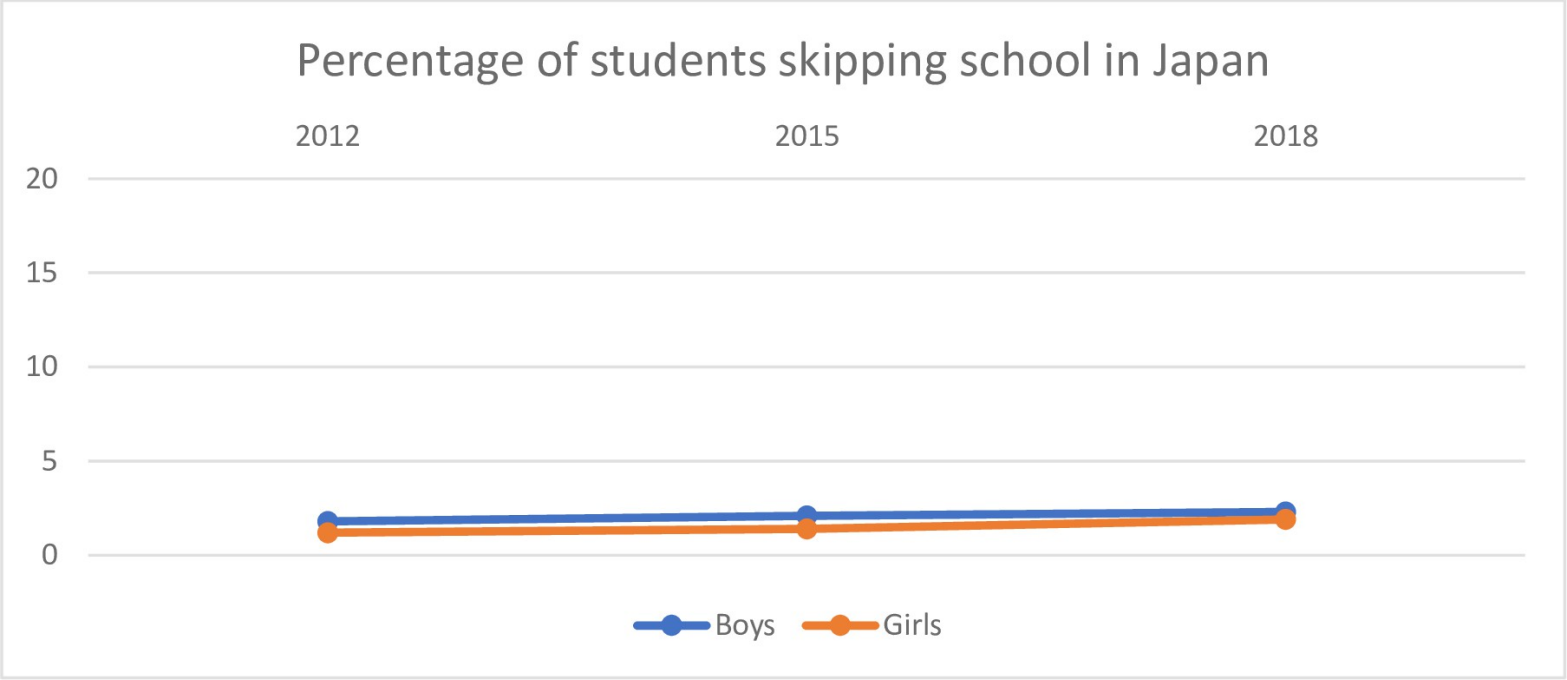
Percentage of boys and girls in Japan who reported that they skipped school.

**Fig 3:**
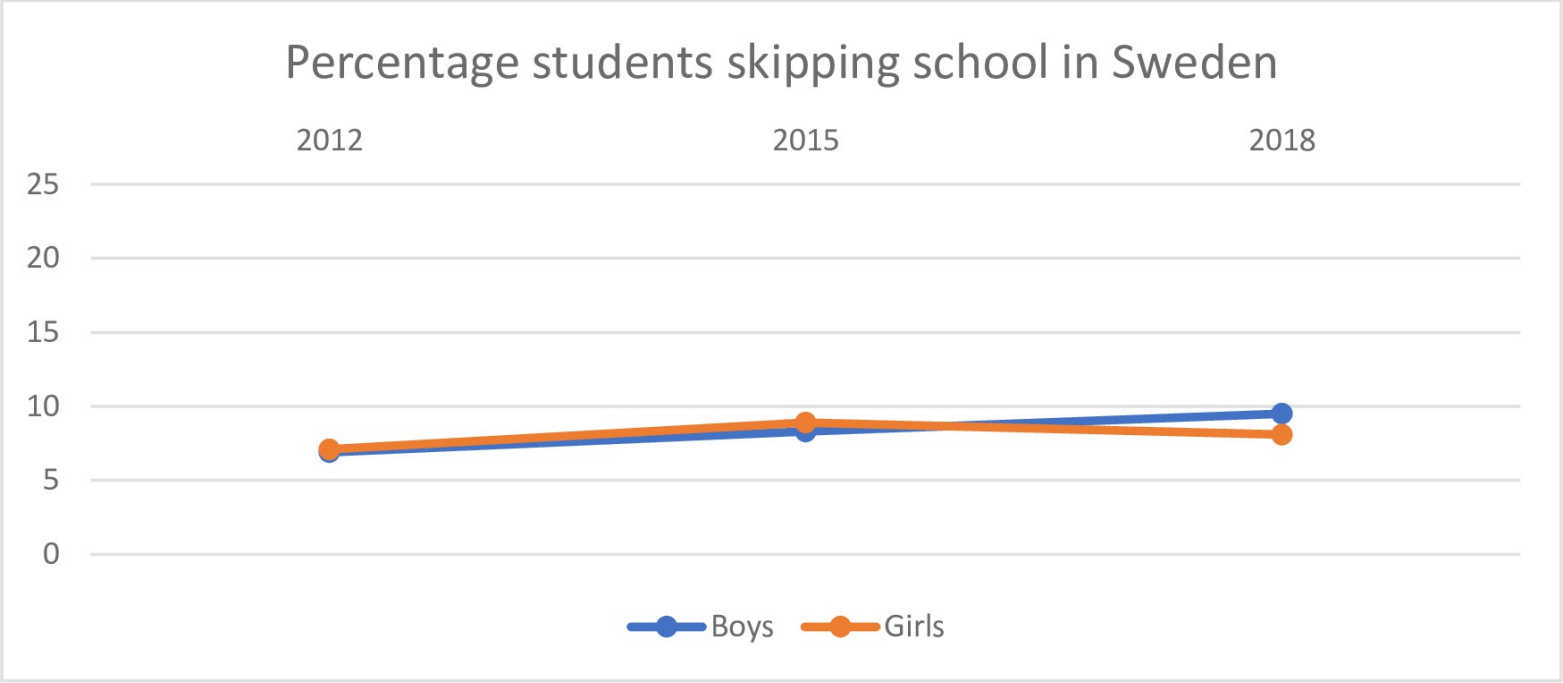
Percentage of boys and girls in Sweden who reported that they skipped school.

**Fig 4:**
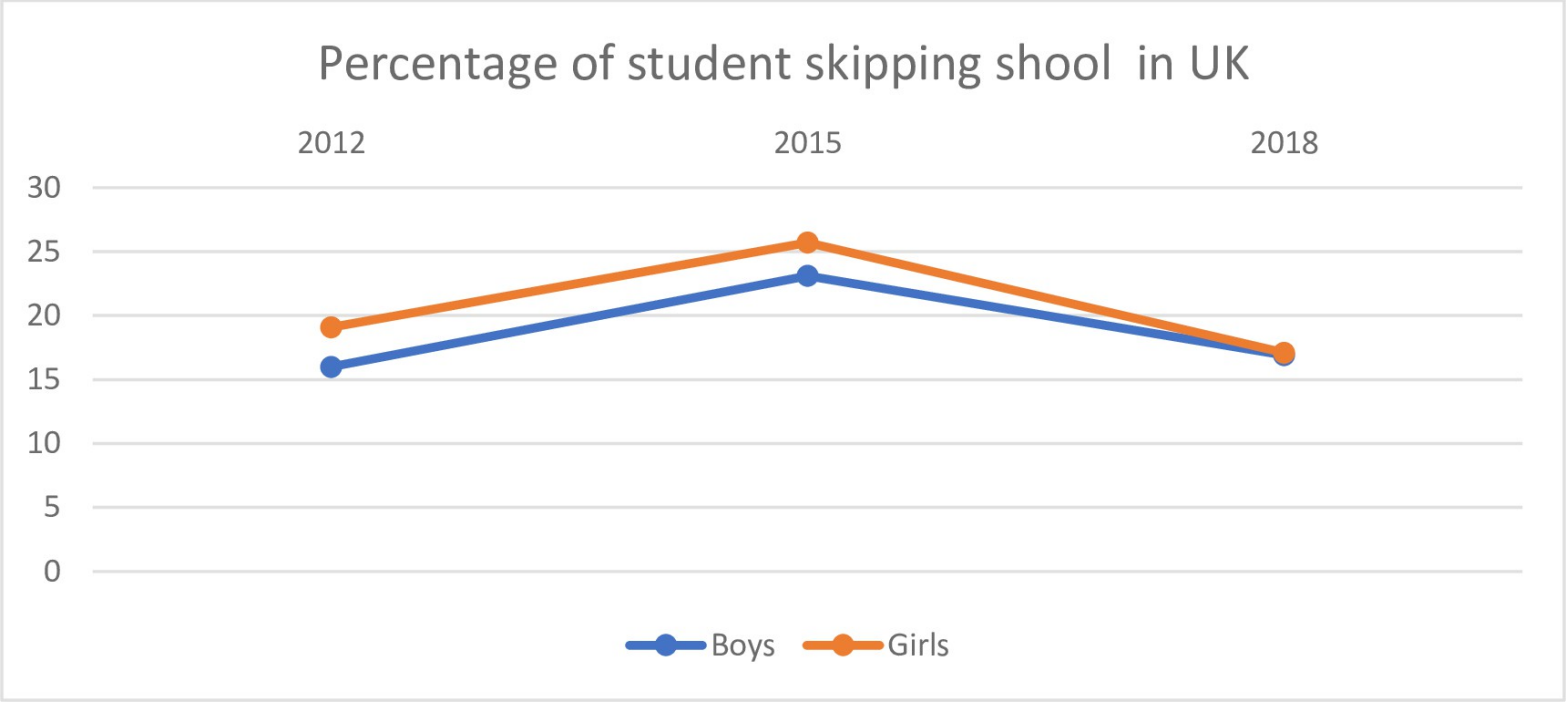
Percentage of boys and girls in the UK who reported that they skipped school.

Figs 1–4 show the percentage of boys and girls who reported that they skipped schools in Germany, Japan, Sweden and the UK as well as the development over the three years 2012, 2015 and 2018. The first figure shows this for Germany.

There are no big differences between boys and girls. In 2012 there was no difference between boys and girls.

Fig 2 shows the percentage of boys and girls who skipped school in Japan.

There is in general a lower percentage of the students who have reported that they have skipped school in Japan than in the other countries. Slightly more boys seem to have reported that they skipped schools than girls, but the difference between boys and girls does not change much over the period.

Fig 3 shows the percentage of boys and girls who skipped school in Sweden.

Generally, there are no big differences between boys and girls in Sweden in 2012 and 2015. In 2018 there seem to be slightly more boys who report that they have skipped school than girls.

Fig 4 shows the percentage of boys and girls who skipped school in the UK.

The percentage of both boys and girls who have skipped school is higher in the UK than in the other countries. There is a difference showing a larger proportion of the girls reporting skipping school than boys in 2012 and 2015, but this difference seems to decrease in 2018.

The tables below show whether there are significant changes between the years in the percentage of boys who have reported that they have skipped school (Table 4) and the same information for girls (Table 5).

**Table 4:** Significance between number of boys skipping school.

	2012–2015	2015–2018	2012–2018
Germany	yes	no	yes
Japan	no	no	no
Sweden	no	no	yes
UK	yes	yes	no

Yes–a significant difference at .05 level between the years

No–no significant difference at .05 level between the year

**Table 5:** Significance between number of girls skipping school.

	2012–2015	2015–2018	2012–2018
Germany	yes	yes	no
Japan	no	no	yes
Sweden	yes	no	no
UK	yes	yes	yes

Yes–a significant difference at .05 level between the years

No–no significant difference at .05 level between the year

There are no significant differences between the number of girls who have reported that they have skipped school in Germany and Sweden between 2012–2018. There is a significant difference between the number of boys who have skipped school between 2012–2018 in Germany and Sweden. No significant differences for boys in Japan. There is a significant difference (p ≤ .05) in the percentage of both boys and girls in the UK who skipped school between 2012–2015, 2015–2018, and when 2012 is compared with 2018.

In general, there are no big differences between boys and girls in respect of how many reported that they have skipped school. The biggest difference is found in the UK in 2012 when 3.1 percent units more girls than boys reported that they had skipped school.

### 6.2. Socio-economic differences

The next area to be explored concerns differences between the students related to their socio-economic status. The students´ socio-economic status is built on the index of social, economic and cultural status (ESCS) which is used in the PISA surveys. This index is based on the students´ answers to questions in the student questionnaire on family and home background. In PISA, a student’s socio-economic status is estimated by the PISA index of economic, social and cultural status (ESCS), a composite measure that combines into a single score the financial, social, cultural and human-capital resources available to students [37]. In practice, it is derived from several variables related to students’ family background that are then grouped into three components: parents’ education, parents’ occupations, and an index summarising a number of home possessions that can be taken as proxies for material wealth or cultural capital, such as possession of a car, the existence of a quiet room to work, access to the Internet, the number of books and other educational resources available in the home” [40, p. 52].

Table 6 shows the students who reported that they had skipped school split into two groups: those in the first quartile and those in the fourth quartile of the PISA index of social, economic and cultural status (ESCS). In the first quartile are the 25% of the students in each country with the lowest scores on the ESCS index. They are from homes where the parents have the lowest level of education and work in professions with the lowest status. They have the smallest number of belongings in their homes. The fourth quartile is the 25% of the students in each country who have the highest scores on the index.

**Table 6:** Number and percentage of students (%) in the first and fourth quartile of the PISA index of social, economic and cultural status (ESCS) who reported that they have skipped school at least one day during the two weeks before they participated in the PISA study.

Country	Germany		Japan		Sweden		UK	
Year	Socio-economic status^1^							
	First quartile	Fourth quartile	First quartile	Fourth quartile	First quartile	Fourth quartile	First quartile	Fourth quartile
	% (n)	% (n)	% (n)	% (n)	% (n)	% (n)	% (n)	% (n)
2012	6.8 (70)	4.0 (41)	2.7 (45)	1.2 (18)	10.9 (127)	4.32 (52)	21.5 (924)	13.4 (397)
2015	9.7 (127)	7.0 (102)	2.4 (39)	1.0 (16)	12.7 (166)	5.5 (74)	29.8 (1,151)	19.4 (712)
2018	8.5 (97)	5.8 (68)	2.6 (40)	2.0 (29)	11.6 (150)	6.4 (86)	22.2 (861)	12.7 (473)

Note. The proportion of students in the first/forth quartile refers to the subset of students in the first/forth quartile who provided responses regarding skipping school for at least one day.

**Fig 5:**
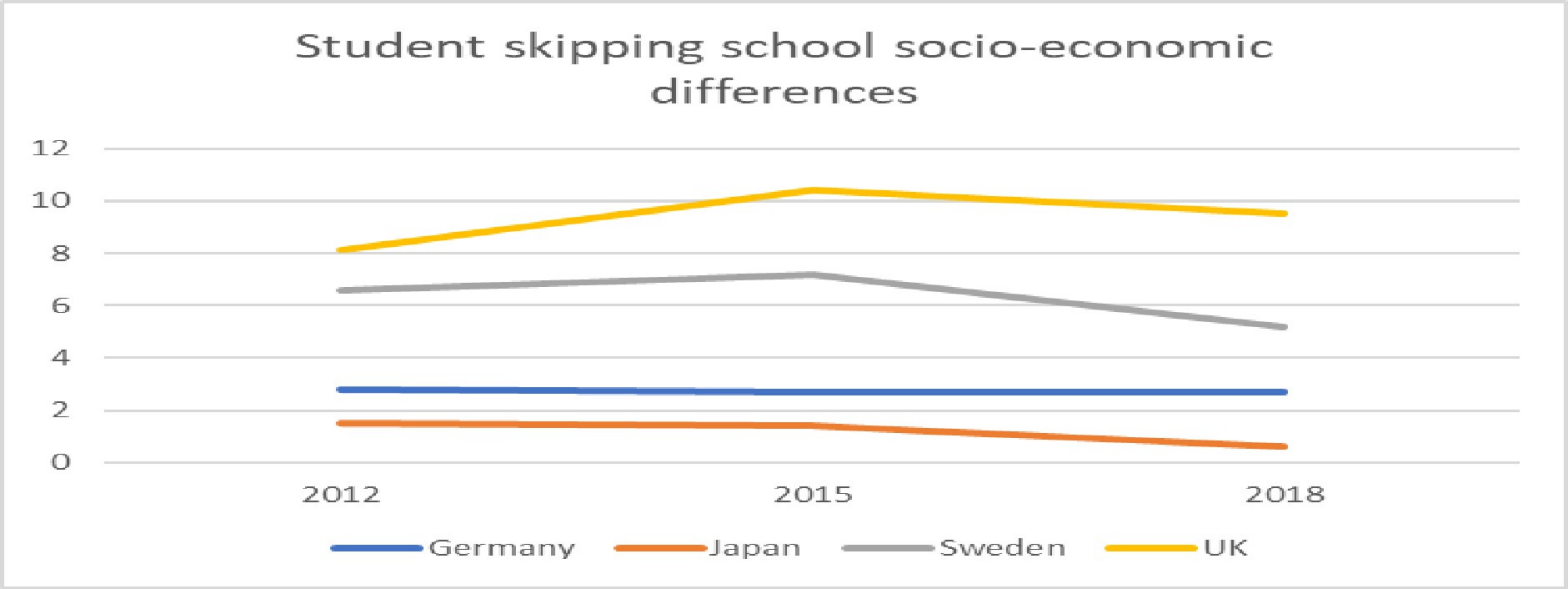
Difference in percent units between students in the first and fourth quartile in the PISA index of economic, social and cultural status (ESCS) who skipped school at least one day.

Table 6 clearly shows that for all three years and in all four countries there is a larger proportion of students from the first quartile of the ESCS index who reported that they have skipped school than from the fourth quartile. To further explore the differences between the countries, the differences in percent units between students in the first quartile and the fourth quartile have been calculated and are presented in Fig 5.

Fig 5 shows that there are larger differences in the share of students in the first and the fourth quartile who have skipped school in the UK than in the other three countries. The socio-economic background seems to have a stronger association with absence in the UK than in other countries. A similar pattern can be seen in Sweden, but the difference between the students from the first and the fourth quartile is smaller compared with the difference in the UK. The socio-economic background seems to have less importance in Germany and Japan.

Tables 7 and 8 show whether the changes in percentages between the years have been significant or not for absent students in ESCS first quartile (Table 7) and absent students in ESCS forth quartile (Table 8).

**Table 7:** Significance between number of students who have skipped school in ESCS first quartile.

	2012–2015	2015–2018	2012–2018
Germany	no	no	no
Japan	no	no	no
Sweden	no	no	no
UK	yes	yes	yes

Yes–a significant difference at .05 level between the years

No–no significant difference at .05 level between the year

**Table 8:** Significance between number of students who have skipped school in ESCS forth quartile.

	2012–2015	2015–2018	2012–2018
Germany	yes	no	no
Japan	no	no	no
Sweden	no	no	no
UK	yes	yes	no

Yes–a significant difference at .05 level between the years

No–no significant difference at .05 level between the year

There have been no significant differences (p ≤ .05) in the percentage of students in the first quartile in Germany who skipped school between the years in the study, while among the students in the fourth quartile, there was a significant difference between 2012–2015. In Japan and Sweden, there have been no significant differences in the percentage of students in the first quartile and the fourth quartile who skipped school between the years in the study. In the UK all the differences in the percentage between the years were significant concerning the students in the first quartile as well as the differences between 2012–2015 and 2015–2018 concerning the students in the fourth quartile.

### 6.3. Migration background

The next issue to explore is the relationship between students’ migration background and skipping school. In PISA, students are grouped into three categories related to their migration background. “Non-immigrant students, who are students whose mother or father (or both) was/were born in the country/economy where the student sat the PISA test, regardless of whether the student him/herself was born in that country or economy. Immigrant students, who are students whose mother and father were born in a country/economy other than that where the student sat the PISA test. Amongst immigrant students, a distinction was made between first- and second-generation students, based on whether the student was born in or outside the country/economy of assessment.

- First-generation immigrant students are foreign-born students whose parents are both foreign-born
- Second-generation immigrant students are students born in the country of assessment but whose parents are both foreign-born” [40, p. 179].

Table 9 shows the share of students referred to as non-immigrants, second-generation immigrants or first-generation immigrants who reported that they skipped school at least one day during the two weeks before they participated in the PISA study.

**Table 9:** Share (%) of non-immigrant, first generation immigrant and second-generation immigrant students who have reported that during the two weeks prior to their participation in the PISA study they skipped school at least one day.

Year	Migration background	Germany	Japan	Sweden	UK
2012	Non-immigrant	4.6	1.5	6.6	17.8
	Second generation	5.3	#	9.1	16.8
	First generation	10.5	#	10.5	18.8
2015	Non-immigrant	6.7	1.7	8	24.8
	Second generation	12.6	#	11.4	23.6
	First generation	19.3	#	12.2	24.3
2018	Non-immigrant	6.1	-	7.6	17.8
	Second generation	9.6	-	12.2	16.0
	First generation	8.9	-	14.6	17.2

There is no available data for immigrant background for Japan 2018

# less than 5 students

Note. The proportion of students in each group refers to the subset of students in each group who provided responses regarding skipping school for at least one day

Table 9 shows that an obviously smaller share of the non-immigrant students reported that they have skipped school than the shares of the first-generation and second-generation immigrant students in Germany and Sweden. The same difference between the non-immigrant students and the first and second generation of immigrant students cannot be seen in the UK. In the case of Japan there are too few students in the categories of first- and second-generation immigrant students in 2012 and 2015 to show a relevant share and from 2018 there are no figures available.

**Fig 6:**
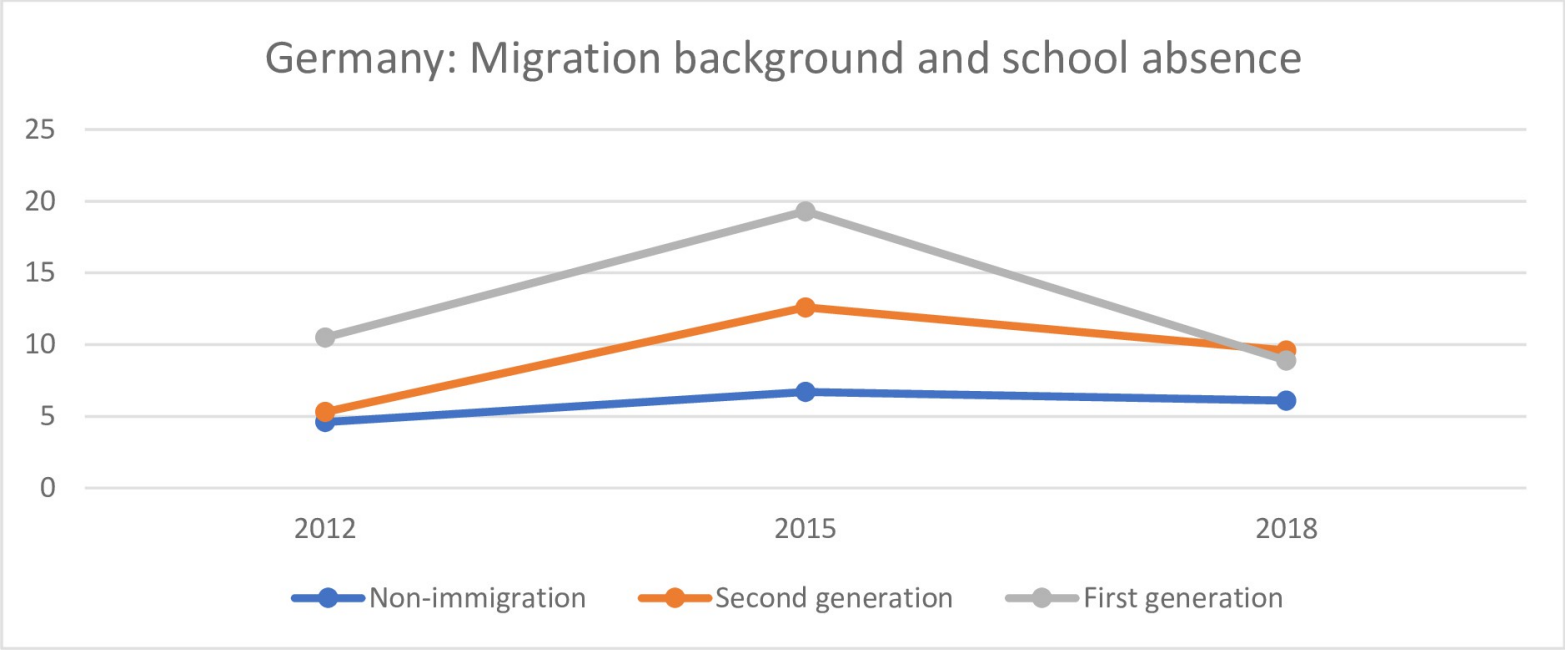
Share of students (%) of non-immigrant, first-generation and second-generation immigrants who reported that during the two weeks prior to their participation in the PISA study, they have skipped school at least one day in Germany.

**Fig 7:**
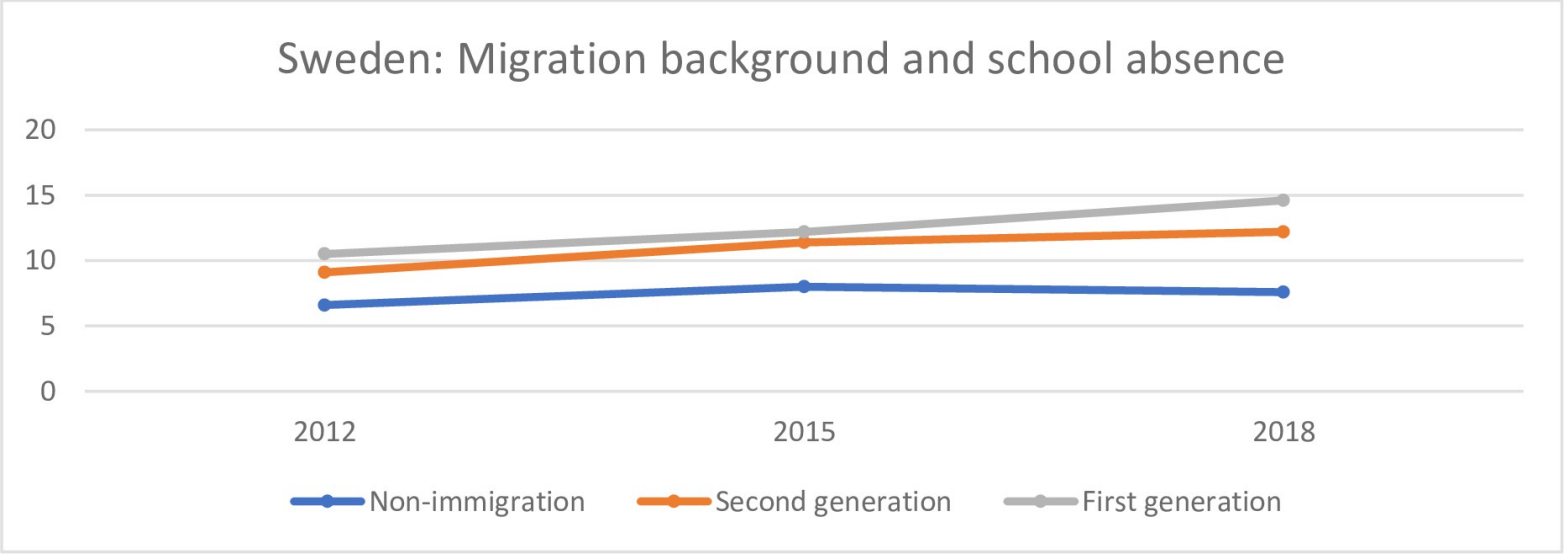
Share of (%) of non-immigrant, first-generation immigrant and second-generation immigrant students who reported that during the two weeks prior to their participation in the PISA study, they skipped school at least one day in Sweden.

**Fig 8:**
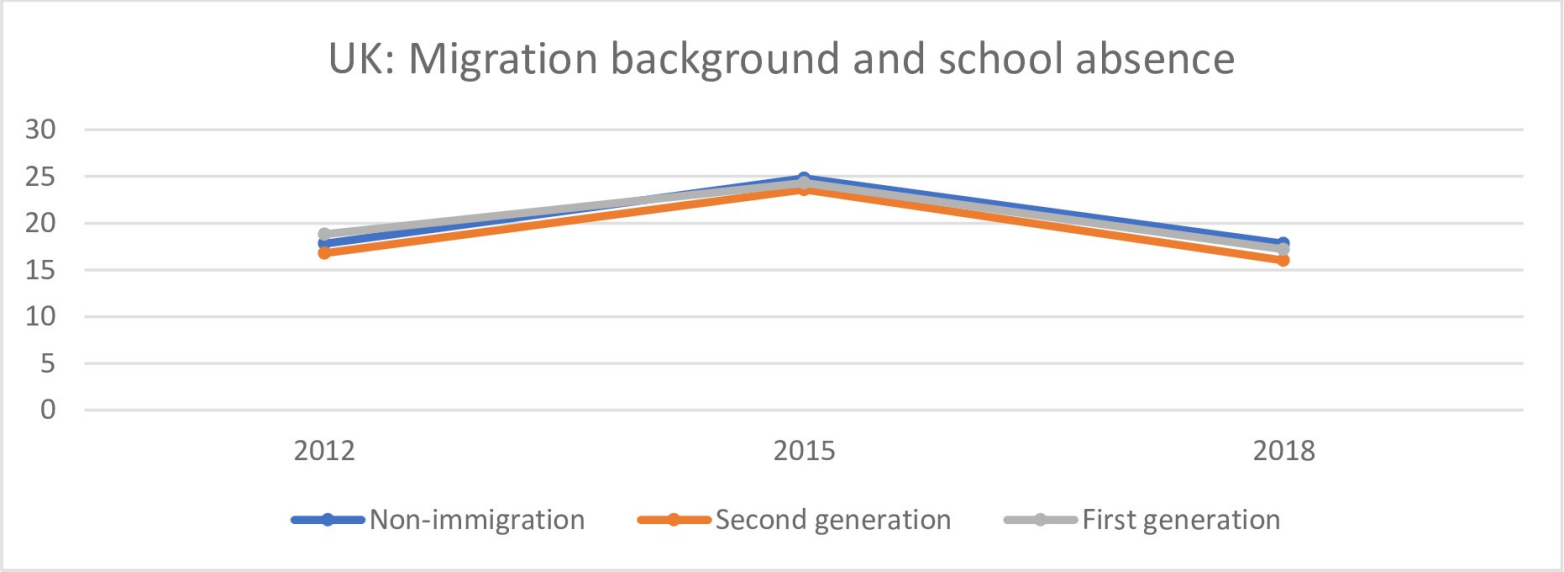
Share (%) of non-immigrant, first-generation immigrant and second-generation immigrant students who have reported that during the two weeks prior to their participation in the PISA study, they have skipped school at least one day in the UK.

Figs 6–8 show the relationship between migration and truancy in Germany, Sweden and the UK. Fig 6 shows the share of students (%) of non-immigrant, first-generation immigrant and second-generation immigrant students who reported that during the two weeks prior to their participation in the PISA study, they skipped school at least one day in Germany.

The first-generation immigrant students seem to have skipped school to a larger degree than the other two groups. For both the first and the second-generation immigrant students there is a larger share in 2015 than in 2012 and 2018. This pattern is more obvious among first-generation students than among the second generation. The share of non-immigrant students who reported that they had skipped school is more or less at the same level during all three years.

Fig 7 shows the share (%) of non-immigrant, first-generation immigrant and second-generation immigrant students who reported that during the two weeks prior to their participation in the PISA study, they skipped school at least one day in Sweden.

As for Germany, there is a lower percentage of non-immigrant students who have reported that they have been absent than among first- and second-generation immigrant students.

Fig 8 shows the share (%) of non-immigrant, first-generation immigrant and second-generation immigrant students who have reported that during the two weeks prior to their participation in the PISA study, they have skipped school at least one day in the UK.

There are no big differences between the three groups in the UK. The three groups also follow the same pattern with a higher share who reported skipping school in 2015 than in 2012 and 2018.

The tables below show whether the changes in percentages between the years of non-migrant students (Table 10), second-generation immigrant students (Table 11) and first-generation immigrant students have been significant or not (Table 12).

**Table 10:** Significance between number of non-migrant students who have skipped school.

	2012–2015	2015–2018	2012–2018
Germany	no	no	no
Japan	no	-	-
Sweden	no	no	no
UK	yes	yes	no

Yes–a significant difference at .05 level between the years

No–no significant difference at .05 level between the year

**Table 11:** Significance between number of second-generation immigrant students who skipped school.

	2012–2015	2015–2018	2012–2018
Germany	yes	no	yes
Japan	no	-	-
Sweden	no	no	no
UK	no	yes	no

Yes–a significant difference at .05 level between the years

No–no significant difference at .05 level between the year

**Table 12:** Significance between number of first-generation immigrant students who have missed school.

	2012–2015	2015–2018	2012–2018
Germany	no	no	no
Japan	no	-	-
Sweden	no	no	no
UK	no	no	no

Yes–a significant difference at .05 level between the years

No–no significant difference at .05 level between the year

There are no significant differences (p ≤ .05) in the percentage of non-immigrant students and first-generation immigrant students in Germany who skipped school between the years of the study. For the second-generation immigrant students, there are significant differences in the percentage of students who missed school between 2012–2015 and 2012–2018.

The absence among all the students with different immigration backgrounds remains at more or less the same level in Sweden over the three PISA studies. There are no significant differences (p ≤ .05) in the percentage for any of the groups between the years in the study.

There are significant differences (p ≤ .05) in the percentage of non-immigrant students who skipped school in the UK between 2012–2015 and 2015–2018. For the second-generation students, there are significant differences between 2015–2018. For first-generation immigrant students there are no significant differences between the years in the study.

## 7. Logistic regression analyses

Logistic regression was performed to ascertain the effects of economic, social, and cultural status (the ECSC-index), immigrant background and gender on the likelihood that students would skip school. The pseudo-R^2^ estimates, Cox & Snell R^2^ and Nagelkerke R^2,^ should be interpreted with caution; they indicate that the models explain almost none or a small share of the variance in the skipping school variable (see Table 13). In Table 14, the coefficients of standard error and p-values are presented. The odds ratio estimates for the significant predictor variables are presented below.

**Table 13:** Logistic regression with a binary output variable for skipping school or not. Skipping school one day or more is coded as 1 and not skipping school is coded as 0.

2012						2015				2018			
		B	SE	Odds ratio	p	B	SE	Odds ratio	p	B	SE	Odds ratio	p
Germany	Intercept	-2,55	0,42	0,08	0,000	-1,48	0,20	0,23	0,000	-2,29	0,24	0,10	0,000
	ESCS	-0,26	0,09	0,77	0,003	-0,09	0,06	0,91	0,124	-0,04	0,06	0,96	0,544
	Native	-0,40	0,41	0,67	0,320	-1,20	0,21	0,30	0,000	-0,34	0,24	0,71	0,161
	Sec. gen.	-0,55	0,46	0,58	0,229	-0,56	0,21	0,57	0,006	0,07	0,25	1,08	0,773
	Female	-0,03	0,14	0,97	0,840	0,14	0,11	1,15	0,179	-0,27	0,12	0,77	0,021
Japan	Intercept	-1,44	1,13	0,24	0,201	-1,08	0,66	0,34	0,100				
	ESCS	-0,60	0,24	0,55	0,013	-0,44	0,15	0,64	0,003				
	Native	-2,72	1,15	0,07	0,018	-2,92	0,68	0,05	0,000				
	Sec. gen.	-0,18	1,39	0,83	0,895	-1,79	1,39	0,17	0,198				
	Female	-0,46	0,22	0,63	0,035	-0,48	0,17	0,62	0,006				
Sweden	Intercept	-2,40	0,29	0,09	0,000	-2,10	0,13	0,12	0,000	-1,82	0,15	0,16	0,000
	ESCS	-0,40	0,08	0,67	0,000	-0,35	0,07	0,71	0,000	-0,20	0,06	0,82	0,000
	Native	-0,18	0,28	0,84	0,526	-0,27	0,15	0,77	0,074	-0,51	0,16	0,60	0,002
	Sec. gen.	-0,04	0,31	0,96	0,903	-0,02	0,16	0,98	0,900	-0,06	0,20	0,94	0,764
	Female	0,04	0,13	1,04	0,774	0,07	0,10	1,07	0,506	-0,16	0,11	0,85	0,126
UK	Intercept	-1,54	0,18	0,21	0,000	-1,17	0,12	0,31	0,000	-1,61	0,12	0,20	0,000
	ESCS	-0,27	0,05	0,76	0,000	-0,28	0,04	0,76	0,000	-0,32	0,04	0,73	0,000
	Native	-0,05	0,18	0,95	0,797	0,03	0,13	1,03	0,828	0,16	0,13	1,17	0,221
	Sec. gen.	-0,19	0,23	0,83	0,410	-0,14	0,16	0,87	0,382	-0,03	0,16	0,97	0,851
	Female	0,22	0,09	1,24	0,013	0,16	0,07	1,17	0,015	-0,01	0,06	0,99	0,930

Note. p values < 0.05 are considered statistically significant

**Table 14:** The model fit statistics.

	2012				2015				2018			
	LL	SE	CSR	NKR	LL	SE	CSR	NKR	LL	SE	CSR	NKR
Germany	226959.57	13608.97	.00	.01	343398.39	14459.3	.01	.03	300503.87	16272.23	.00	.01
Japan	165009.11	18739.20	.01	.04	187342.39	19594.91	0	.03	omitted			
Sweden	45579.42	2069.22	.01	.02	50704.37	2242.14	.01	.02	51924.42	2191.62	.01	.02
UK	613353.70	14197.56	.01	.01	636845.49	13290.25	.01	.02	493699.76	9401.98	.01	.02

Note. Log Likelihood (LL), Cox & Snell R^2^ (CSR), Nagelkerke R^2^ (NKR)

In PISA 2012, the likelihood of skipping school decreases if the students have higher ESCS in all four countries. The odds ratio is highest in Germany (.77) and lowest in Japan (.55). In Japan, the native students had a slightly higher likelihood (.07) to not skip school than the first- and second-generation immigrant students. Finally, in the UK the girls were 1.24 more likely to not skip school than the boys.

In PISA 2015, the non-immigration students in Germany were .30 and the second-generation immigrant students were .57 more likely to not skip school than the first-generation immigrant students. The non-immigration students in Japan were slightly more likely (.05) not to skip school than the first- and second-generation immigrant students. The Japanese female students (.62) and the students with higher ESCS (.64) were less likely to skip school than the male students and students with lower ESCS. In Sweden (.71) and the UK (.76), the likelihood of skipping school decreased if the students had a higher ESCS. In the UK the female students were 1.17 less likely to skip school than the boys.

In PISA 2018 the data for immigrant backgrounds were omitted in Japan. Girls in Germany were .77 less likely to skip school than boys. In Sweden and UK, the likelihood of skipping school decreases if the students have higher ESCS. Also, in Sweden the native students are .6 less likely to skip school than first- and second-generation students.

In sum, although the models explain a small variance in the skipping school variable, it can be noted that the economic, social, and cultural status seems to be related to skipping school in all three years in Sweden and UK. In Japan, being a native student decreases the risk of skipping school in 2012 and 2015. Unfortunately, these data are omitted for Japan in PISA 2018. The other predictor variables have no consistent pattern over the years and countries.

## 8. Sociodemographic factors and students’ absence from school

This article has explored the sociodemographic factors of gender, socio-economic background and migration background in relation to self-reported absence from school in Germany, Japan, Sweden and the UK. The study investigated which students reported that they had skipped school at least one day in the two weeks prior to the PISA test. In the following, we will discuss how the results from the study can be interpreted in relation to earlier studies.

On average, across OECD countries in 2018, 21 percent of students reported that they had skipped at least one day of school in the two weeks prior to the PISA test [41]. In this study, we have focused on four countries with different school and welfare systems and cultural backgrounds. We have used data from the years 2012, 2015 and 2018. We can see a rather large variation in the percentages of students who report that they have skipped school at least one day in the four countries. The percentage of students varies from 24.4 percent in the UK in 2015 to 1.5 percent in Japan in 2012. Generally, the UK seems to have the highest percentage of students who reported that they skipped school among the four counties (see also Fredriksson, et al., [11]). The countries included in this study have a lower proportion of students who reported that they had skipped school in 2018 than the average among the OECD countries [41]. Generally, the UK stands out as having a higher percentage of students who have reported absence than the other countries. This does not seem to be related to any specific group of students. Regardless of which groups of students were analysed, the percentage among the students from the UK is higher than in the other three countries. At the same time, figures for self-reported absence in Japan are much lower. Reasons for that need to be further explored. Zhang et al. [42] suggest that students may try to hide an absence from parents or teachers and be reluctant to report their own absence in order to avoid social consequences (as cited in Keppens et al. [43, p. 3]). Such behaviour patterns can be culturally dependent. Hence, respondents are more likely to conceal or fail to recall their truancy out of fear of the consequences, resulting in an underestimation of the actual truancy rate. The numbers for Germany and Sweden are somewhere in between. It becomes obvious that there are–as we expected–differences between the absence rates in different countries and it will be interesting to study more in detail how this can be related to the education systems, welfare state models, or other aspects that can be relevant for students’ school attendance. Is it possible, based on figures from the PISA studies, to identify some characteristics of the students who reported that they have skipped school in the four countries?

### 8.1. Gender

There do not seem to be any obvious differences between boys and girls in respect of how they reported that they have skipped school, which seems to be in accordance with earlier research (see Reid [27]; Maynard et al. [3]: Keppens & Spruyt [10]). Our study contributes to the divergent information on the role gender plays in absence. There is no consistent pattern over the years and in countries related to the likelihood for boys or girls to skip school. The percentual differences in all countries and in all years between absent and non-absent girls and absent and non-absent boys are significant (p ≤ .05), There are significant differences (p ≤ .05) in the percentage of girls who missed school in Germany between 2012–2015 and between 2015–2018, in the UK between 2012–2015, 2015–2018 and 2012–2018. There are significant differences (p ≤ .05) in the percentage of boys who missed school in Germany between 2012–2015 and between 2012–2018, in Sweden between 2012–2018 and in the UK between 2012–2015 and between 2015–2018.

We expected that we would find a variation of different patterns between boys and girls in the studied countries, in line with that results on gender patterns related to school absence are inconsistent according to the international literature. This expectation proved to be true, and we can point out a number of interesting impressions from our statistical analysis.

The regression analysis showed that in the UK the boys were 1.24 more likely to skip school than the girls in 2012. However, differences that were visible between boys and girls in the studies from 2012 and 2015 had diminished in the data from 2018. Japan is a country where slightly more boys than girls report skipping school. This is in line with early studies on gender and truancy, where boys were reported to be more truant [19, 26]. In both Sweden and Germany, differences have changed from equal shares in 2012 and slightly higher shares of girls in 2015 to slightly more boys reporting truancy than girls in 2018. There are factors we have not studied that may influence patterns of answering a question about skipping school. The student populations in all four countries can be assumed to be equally balanced between boys and girls We cannot exclude that there are different social and cultural factors that influence the social reality of boys and girls in school and their patterns of answering the questions as gender is a very complex dimension. The observed changes under time for gender patterns are asynchronous between the studied countries, except for Sweden and Germany, where the development seemed to follow a similar pattern.

### 8.2. Socio-economic status

In all countries in all the years the proportion of students who report that they skipped school are higher in the first quartile of the ESCS index than in the fourth quartile This is in line with earlier research. Students who grow up in affluent homes seem to be at a lesser degree at risk of missing parts of their compulsory schooling than students with the least favourable home background. The differences in self-reported absence between the students in the first and in fourth quartile are clearly larger in the UK than in Germany and Japan. The differences in Sweden are also larger than in Germany and Japan, but the gap between the two groups has diminished between 2015 and 2018. The economic, social, and cultural status is related to skipping school in all three years in Sweden and the UK. The percentual differences (p ≤ .05) in all countries and in all years between absent and non-absent students in the first ESCS quartile and the fourth quartile are significant. There are significant differences in the percentage of students in the fourth quartile in Germany between 2012–2015. In the UK all the differences in the percentage between the years were significant concerning the students in the first quartile as well as the differences between 2012–2015 and 2015–2018 concerning the students in the fourth quartile.

The results are generally in line with our expectation that socio-economic background is likely to influence absence in different countries as we know from a rich research literature, mainly from the field of educational sociology, but also from educational psychology, that SES influences many aspects of education. Our results add to earlier studies by for example Maynard et al. [3] and Vaughn et al. [21] who showed the relevance of SES for truancy in the US. Our study also differentiates what Keppens and Spruyt [10] have illustrated for European countries and adds to the knowledge that the effect that socio-economic differences may have in different countries on truancy can vary. Of interest in this context is to look at the values of the four countries on the Gini coefficient, which is an index for the degree of inequality in the distribution of income, often used to estimate how far a country’s income distribution diverges from an equal distribution. The Gini coefficient can range from 0, which would indicate complete equality, to 1, which would indicate complete inequality. According to statistics from OECD [44] the Gini coefficient for disposable income after taxes and transfers is among the four countries highest in the UK (0.366), followed by Japan (0.334). Germany (0.296) and Sweden (0.276) have lower values. It can be noted that even though the UK and Japan have the highest values on the Gini coefficient we also find the largest differences among the four countries between them when we look at the differences between the share of students in the first and the fourth quartile who have skipped school. The UK has larger differences than the other three countries and Japan has the smallest differences. It seems that inequalities in societies may not be the only factor that influences students´ truancy. How the structures of education systems and welfare systems can influence the impact of students´ socio-economic status on truancy is something that would be relevant to further research.

### 8.3. Migration

In Sweden and Germany there is a higher proportion of students with a first-generation or second-generation immigration background who reported that they skipped school than among the non-immigrant students. In Japan, there were too few students with a first-generation or second-generation immigration background to make comparisons possible, but the Japanese data available shows that being a native student decreases the risk of skipping school in 2012 and 2015. In the UK the differences between immigrant and non-immigrant groups reporting school absence were smaller than in Sweden and Germany. The percentual differences in all the countries and in all the years between absent and non-absent non-immigrant students are significant (p ≤ .05). The percentual differences in all countries and in all the years between absent and non-absent second-generation students are significant. The percentual differences in all the countries and in all the years between absent and non-absent first-generation students are significant.

As we know from other comparisons between non-migrant and migrant students, those who have lived longer in their respective countries seem to do better in school. That means that our expectation that this will also be reflected in the absence rates is confirmed, but the observed patterns are different in the four studied countries.

The share of immigrants in the population of OECD countries averaged 12% in 2015. Sweden, (20%) Germany (17%) and the UK (15.5%) all had a higher share of immigrants than the OECD average, while Japan, with 1.5%, had a much smaller share [45]. Taking the low share of immigrants in Japan into consideration it is no surprise that there were no figures on the percentage of migrant students in PISA available for Japan. Although, Sweden, Germany and the UK have higher shares of immigrants than the OECD average it could also be noted that they have a lower proportion of students who reported that they had skipped school in 2018 than the average among the OECD countries [40]. A high percentage of immigrants in a country may not necessarily be reflected in higher truancy numbers.

We note that in the US context ethnicity/race has often been used as a variable. As that type of data is not available in the PISA studies it has not been possible to look at this aspect. Keppens and Spruyt [10] mention in their study differences with regard to ethnicity. However, as this study also builds on PISA-data, ethnicity seems to be used as an equivalent for migration background. We chose to stay with the term migration background in our study. Children with a migration background can have very different ethnical backgrounds. From a Swedish perspective, we think that the term ethnicity should be handled with care and each school system needs to take decisions on which categories are useful for their analysis. Official school attendance statistics in England analyse school attendance in relation to other background variables that are regarded as relevant to catch social inequalities, such as free school meal eligibility, first language and ethnic group [46]. There may be reasons why they did not choose migration background as a central category. Although migration background can give some information about ethnicity it should not be compared with studies where ethnicity/race has been used as a variable. Generally, it can be assumed that among immigrant students there are not only differences between first- and second-generation immigrant students but also between immigrant students from different countries and with different reasons for their migration. It should be kept in mind that migration and integration policies and migration history are very different between the countries included in this study.

### 8.4. Developments over time

Finally, when the percentages of absent students are compared over time and in different countries it is difficult to find any trends. This may be in line with the findings in the US by Maynard et al. [3] who reported that school attendance behaviour was difficult to influence.

There are only consistently significant differences in the figures for all years in the UK for students in the first quartile of the ESCS index. There are no other consistent changes for any group in any of the countries that include significant differences in the figures for all the years included. However, for some groups in some countries during some years there are significant differences in the percentage of absent students. Developments over time show for example, that there were significant differences in Germany for the second-generation immigration students in the percentage of students who missed school between 2012–2015 and 2012–2018. Migration background seems to have lost some explanation value while socio-economic differences were clearer reflected in the data. Trends were however somewhat opposite in Sweden. Such numbers and correlations need to be interpreted with care. As the figures between the years vary from study to study and several curves are shaped with a peak in 2015, it could be relevant to just look at the differences between 2012 and 2018. The difference in numbers between those years is significant for boys in Germany and Sweden, girls in Japan and UK and students in the ESCS first quartile in the UK and second-generation students in Germany.

Although there are differences between the years, there are no obvious trends. So far, the assumption that changes would be small can be confirmed. With only three consecutive studies where an identical question on skipping school was asked, it is doubtful whether any of these observations can be considered trends. The time between 2012 and 2018, maybe too short a time span to see trends, even if in some cases, the differences proved interesting insights. If PISA data will include the question on absence in the future, it will be interesting to follow new developments. Although, we can for the next data set expect changes due to the COVID-19 pandemic, which most likely influenced school attendance strongly.

## 9. Limitations

There are some limitations related to the use of PISA data in this context that have to be acknowledged and which have been noticed in earlier studies using PISA data (Fredriksson et al., [11]).

Students who reported in PISA that they had been absent from school one day or more in the two weeks before they completed the PISA questionnaire may not be the students who have been absent from school for longer periods. Students who are in the process of increasing their absence, may correspond to what was mentioned above as problematic absence from schools, and a matter of such, absenteeism that will have a negative impact on the students´ possibility to develop cognitively and socially to fulfil the aims of education [47].

The information in PISA on absence is based on the students´ self-reporting in the student questionnaire. As the students complete the questionnaire in school it cannot be excluded that there are students who have reasons to overstate or understate their absence. It can be assumed that in a large-scale study, such as PISA, tendencies to overstate or understate may balance each other, but there can be systematic pattern of under- or overestimation [10].

PISA is a study based on randomly selected national samples of 15-year-old students. There is always a risk that a sample is not fully representative of the population it is supposed to represent. In the case of PISA this risk is generally estimated to be about five percent [35, 48]. Obviously, only a small group of the students who have participated in the PISA study are students with school attendance problems. When a representative sample of all 15-year-old students in a country is used to describe a smaller group of students within the whole population of 15-year old students there is a risk that this smaller group is not representative of the corresponding group in the whole population, even if the sample is representative of the whole population in general.

## 10. Conclusions

The most obvious conclusion that can be drawn from the analysis of the characteristics of the students who skipped school is that students´ self-reports of school attendance vary in different countries and to a certain extent with regard to sociodemographic factors.

Absence seems to be more related to socio-economic background than the other two variables explored. Results on gender are not consistent between countries or time of study. In Sweden and Germany, there is a lower percentage of non-immigrant students who report that they have skipped school than first- and second-generation immigrant students, but numbers are changing between years. In the UK the figures are more ambiguous. As we did find differences between patterns of school attendance in different school systems, there may be also different and more or less successful ways to meet school attendance problems, which should be explored more in detail.

The differences we found for different student populations need to be further investigated taking national context and differences in school and welfare systems into account. We also note that the PISA questionnaires contain more information that could be used for further analyses of student absenteeism in relation to different student characteristics, opinions and results.

PISA has been designed with the goal to study student achievement, but has also the potential to make it possible to follow developments in school attendance. This may be specifically important as no international comparative data on school attendance problems are available.

## Supporting information

S1 Appendix Question about truancy in four languages. (DOCX)
