# Supplementary figures and images for: Which students skip school? A comparative study of sociodemographic factors and student absenteeism using PISA data
Source: PLoS One. 2024 May 22;19(5):e0300537. doi: 10.1371/journal.pone.0300537 (PMC11111071; doi:10.1371/journal.pone.0300537)

**Appendix A**

**Question about truancy in four languages:**

**English**


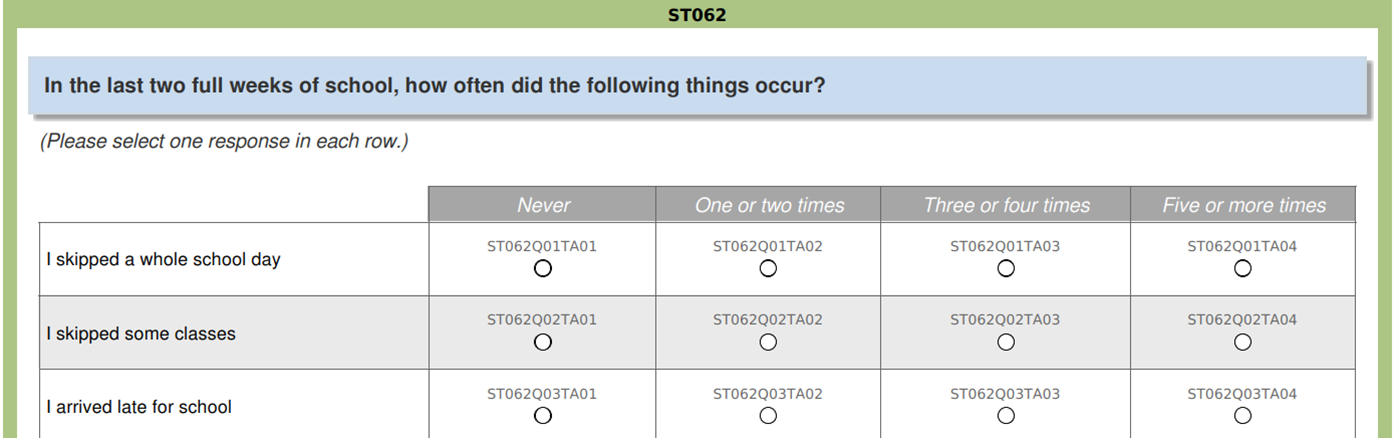


**German**


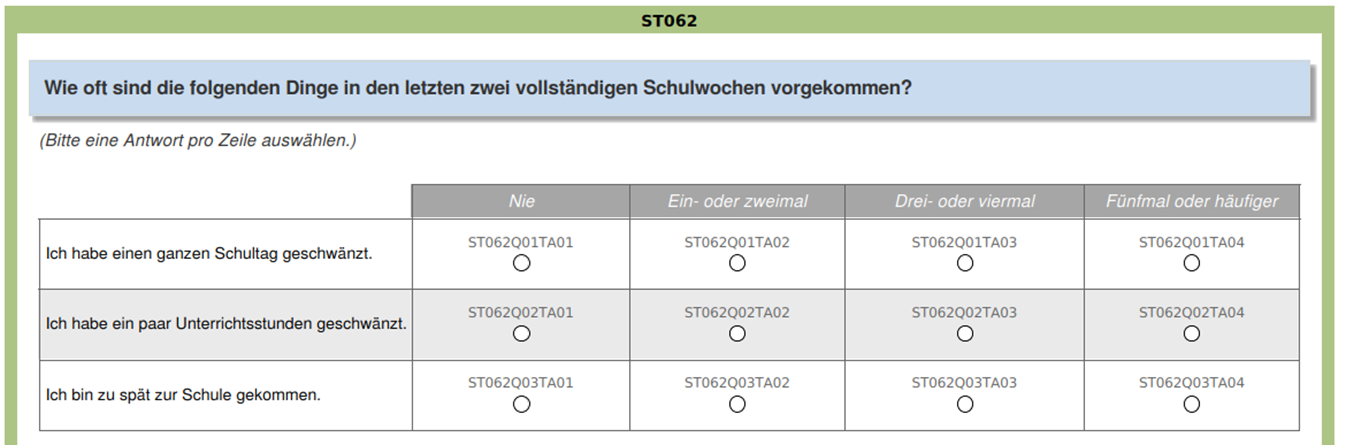


**Japanese**


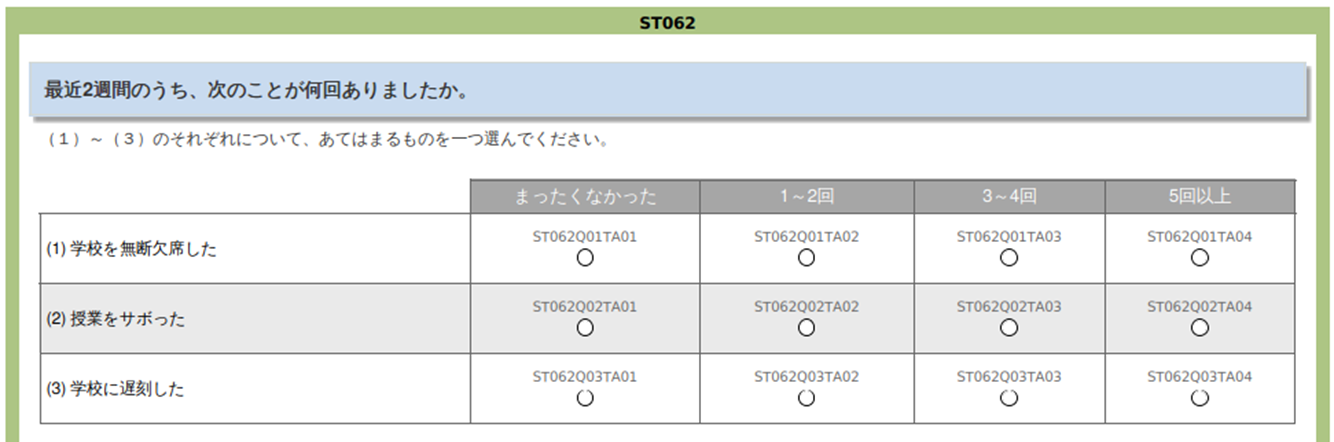


**Swedish**


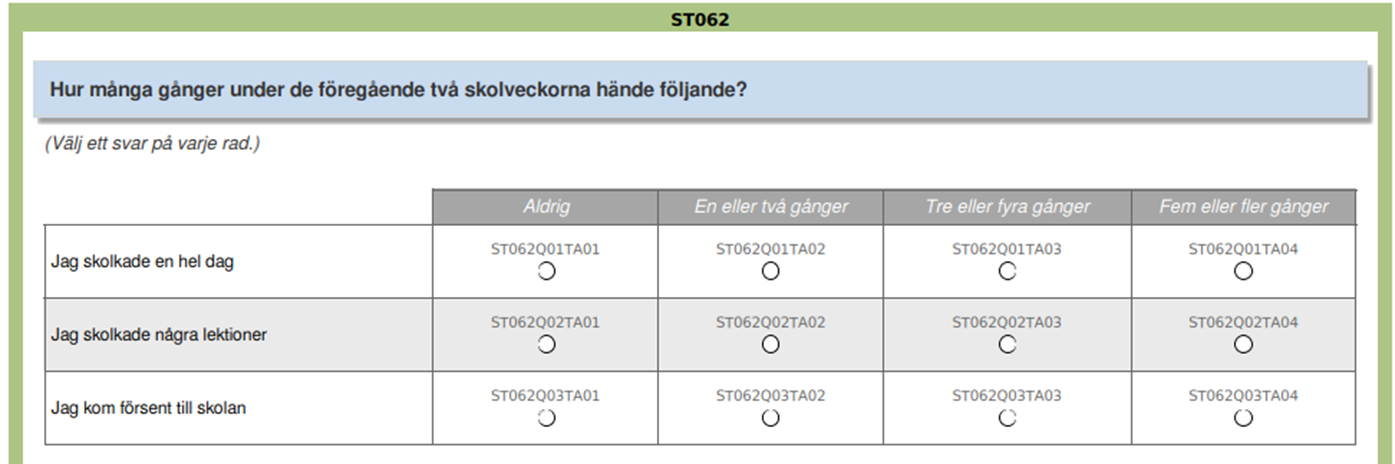

Supplement: S1 Appendix — (DOCX) [file pone.0300537.s001.docx]
